# A Rare Case of Isolated Spontaneous Common Carotid Artery Dissection Treated by Telescoping Stents

**DOI:** 10.7759/cureus.46933

**Published:** 2023-10-12

**Authors:** Yoon-Soo Lee

**Affiliations:** 1 Neurosurgery, Daegu Fatima Hospital, Daegu, KOR

**Keywords:** carotid stent, telescoping, dissection, common carotid artery, spontaneous, isolated

## Abstract

An isolated spontaneous common carotid artery (CCA) dissection is an extremely rare cause of stroke, and standard treatment guidelines are not yet established. This case report presents a rare case of isolated spontaneous CCA dissection but with typical and obvious radiological findings, and it could be informative and educational to clinicians. Telescoping multiple carotid stents can be a feasible treatment option for this case with recurrent ischemia due to a long segment dissection.

## Introduction

Spontaneous carotid dissection can be an important cause of stroke usually in the young population, and it is encountered usually in the cervical segment of the internal carotid artery (ICA). Spontaneous dissection of the common carotid artery (CCA) is an extremely rare cause of ischemic stroke [1-6]. Most CCA dissections are caused by an extension of an aortic dissection, and direct traumas and iatrogenic etiologies, such as vascular procedures, are known to be relatively less common causes [1,7-11]. However, a purely isolated spontaneous CCA dissection is extremely rare, and only a few cases are reported in the literature. Its etiology is not yet known. When the continuity of the intima and media is disrupted, thrombus formation occurs in the false lumen. It can lead to a stenosis of the true lumen, causing hemodynamic infarction. In other cases, the local thrombus within the lesion can be a significant source of embolism, causing multiple embolic infarctions or even intracranial large vessel occlusions.

Due to the extremely low incidence of the isolated spontaneous CCA dissection, there exist no evidence-based guidelines for its treatment. According to reports on spontaneous cervical ICA dissection, a medical treatment could be the standard for patients who promptly respond to anticoagulation or antiplatelet therapy, but an emergent endovascular recanalization may be required for those with a concomitant intracranial large vessel occlusion and endovascular stenting for those with a significant stenosis causing recurrent ischemic attacks even after the best medical treatment [6,12,13]. Similar strategies could be applied to the treatment of isolated spontaneous CCA dissection.

Herein, the author reports on a rare case of isolated spontaneous CCA dissection and describes the course of diagnosis and treatment.

## Case presentation

**Figure 1:**
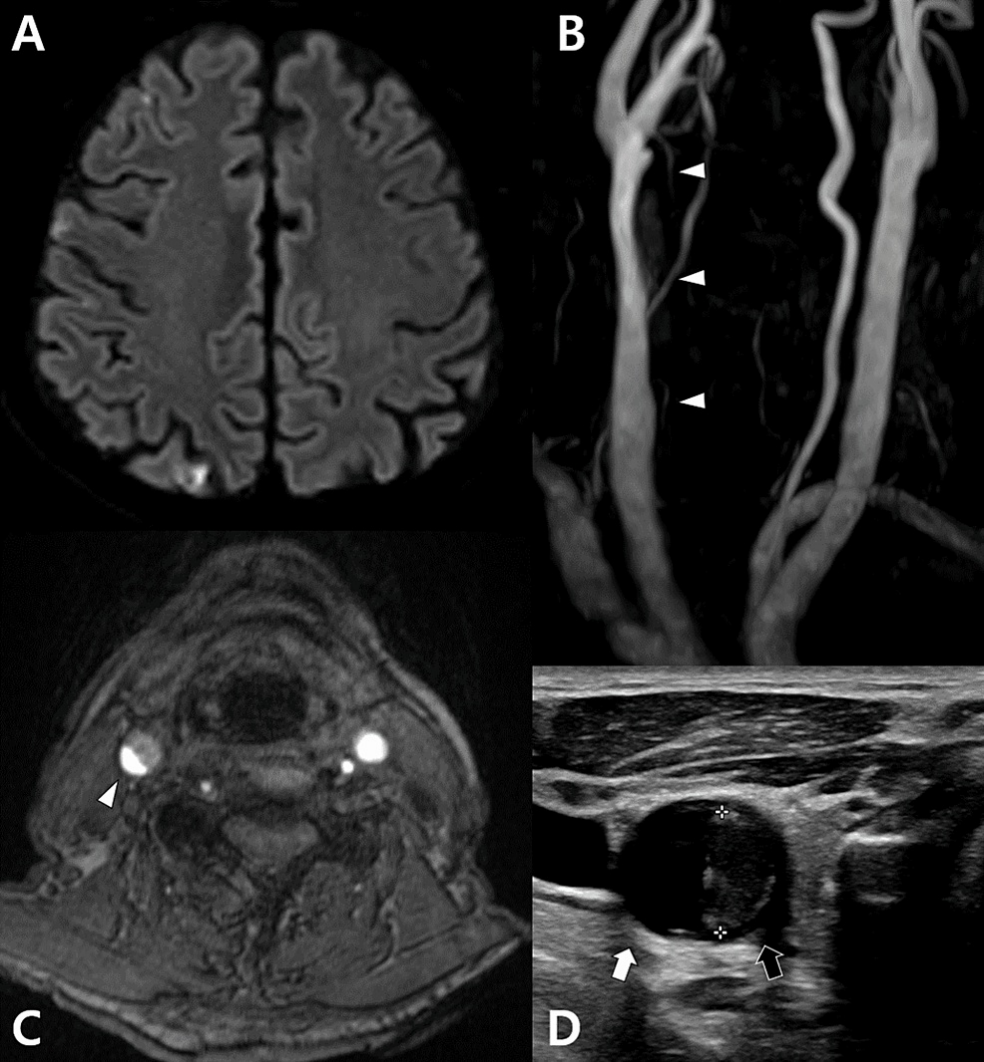
Initial radiological findings (A) Diffusion-weighted magnetic resonance imaging reveals multi-focal infarctions on the right hemisphere. (B, C) Neck magnetic resonance angiogram and its source image show a moderate stenosis (white arrowheads) from the right mid to distal common carotid artery. (D). The cross section of carotid ultrasonography shows a lumen (white arrow) in a semilunar shape and thrombus (black arrow) in the remaining semilunar portion.

An 83-year-old male presented with abrupt dysarthria and transient left hemiparesis. The patient was under regular medication for hypertension and hyperlipidemia. He was admitted to the department of neurology. The magnetic resonance (MR) imaging revealed multi-focal infarctions on the right hemisphere (Figure 1A). A moderate stenosis was seen from the right mid to distal CCA on the neck MR angiogram and its source image (Figure 1B, 1C). The cross section of the carotid ultrasonography showed a lumen in a semilunar shape and thrombus in the remaining semilunar portion (Figure 1D). The carotid ultrasonography six months previously had not demonstrated such findings. An echocardiogram revealed nonspecific findings.

**Figure 2:**
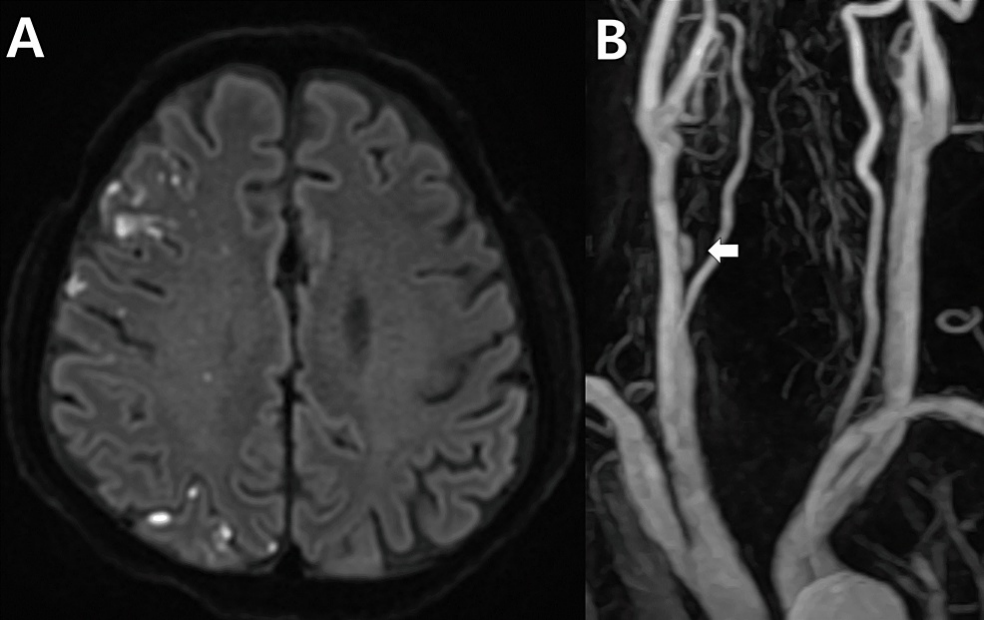
Follow-up radiological findings (A) Diffusion-weighted magnetic resonance imaging on the fourth day shows an increased number of multi-focal infarctions. (B) Neck magnetic resonance angiogram reveals a newly developed flow (white arrow) in the false lumen. Note the dynamic and unstable changes in the morphology of the mural hematoma when compared to the initial neck magnetic resonance angiogram.

The MR imaging on the fourth day showed an increased number of multi-focal infarctions (Figure 2A). The neck MR angiogram showed a newly developed flow in the false lumen where preexisting thrombus had probably migrated away (Figure 2B). The overall findings suggested the presence of a long-segment spontaneous CCA dissection with dynamic and unstable changes in the morphology of the mural hematoma.

**Figure 3:**
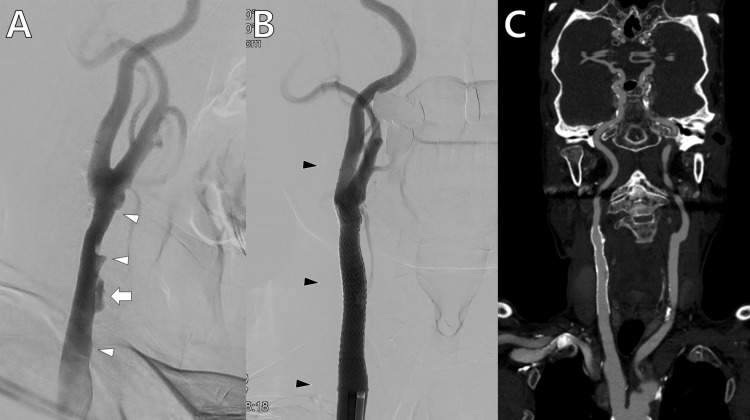
Intra- and post-operative radiological findings (A) Carotid angiogram showed an irregular intimal flap (white arrowheads) with the false lumen (white arrow) and the remaining mural hematoma. (B) Stenting (black arrowheads) is performed using two stents in a telescoping configuration with an overlapping at the mid portion. Note that the false lumen is completely collapsed and the true lumen is fully enlarged. (C) Coronal section of the head-neck computed tomography angiography a week later shows a patent flow within the carotid stents.

The patient was transferred to the department of neurosurgery. The carotid angiogram showed an irregular intimal flap with the false lumen and the remaining mural hematoma (Figure 3A). The North American Symptomatic Carotid Endarterectomy Trial ratio was 69.83%. Due to the progression of embolic events even after strict medical managements, a carotid stenting was planned. Under local anesthesia, an 8 Fr Asahi Fubuki guiding catheter (Asahi Intecc Co. Ltd., Aichi, Japan) was located in the right proximal CCA. A 6 mm Spider FX embolic protection device (Medtronic, Minneapolis, MN, USA) was installed at the cervical ICA for distal protection. A balloon angioplasty was omitted because the mural hematoma was regarded to be soft, and a stabilization of the intimal flap was the main purpose of the procedure. A 10×40 mm Precise PRO RX stent (Cordis, Miami, FL, USA) was installed at the proximal ICA to the distal CCA, and another identical stent was installed at the distal to mid CCA. The stenting was performed in a telescoping configuration with an overlapping at the mid portion, and the long segment lesion, including adequate safety zones on each side, was fully covered by the stents. The false lumen was completely collapsed, and the true lumen was fully enlarged without remaining stenosis (Figure 3B). The patency of the carotid stents was evidenced by a head-neck computed tomography angiography a week later (Figure 3C). The patient gradually made a full recovery without any neurological deficits.

## Discussion

Although a purely isolated spontaneous CCA dissection is extremely rare with less than 30 cases described in the literature, it is crucial to rule out its presence for the management of ischemic stroke patients because most of these reported cases were symptomatic [6]. However, due to its low incidence and absence of a co-existing aortic dissection or a history of direct trauma, clinicians tend to underestimate its presence, and it is frequently misdiagnosed. The findings of dissection, such as an intimal flap and mural hematoma, could be veiled by the collapsed true lumen, and it may be difficult to notice initially and immediately. Moreover, it could be difficult to differentiate an irregular atherosclerotic plaque in the CCA from a dissection just by evaluating an angiographical morphology. A carotid ultrasonography can be useful in detecting the intimal flap, confirming the range of dissection, and differentiating from a calcified plaque [4]. The clinical course should be monitored carefully with detailed inspections of imaging for suspicious lesions because a spontaneous CCA dissection often leads to unstable stenosis causing recurrent embolism. Clinical aggravation from recurrent embolic events with dynamic radiographical change in the configuration of the stenosis, as in this case study, can be a clue for the diagnosis of a spontaneous CCA dissection. Although the source image of the neck MR angiogram and the cross section of the carotid ultrasonography showed the typical findings of a dissection in this case study, neither the neurologist nor the radiologist had the impression of a spontaneous CCA dissection initially and regarded it as a usual carotid stenosis until a clinical suspicion was raised by the neurosurgeon.

The pathophysiology for isolated spontaneous CCA dissection is not well known but it seems to be multifactorial, including familial and genetic predispositions and other risk factors, such as hypertension, hyperlipidemia, infections, and uncommon connective tissue diseases [3,6]. In the current case, the patient was already diagnosed with hypertension and hyperlipidemia, but the CCA dissection occurred spontaneously without any traumatic events even after regular medications and work-ups on the cardiac status.

Although there are no clear evidence-based guidelines for the treatment of the spontaneous CCA dissection, strategies for the treatment of the cervical ICA dissection could be similarly adopted [6,12,13]. The treatment goal should be the maintenance of adequate distal flow for cerebral perfusion, the prevention of further distal embolism, and the prevention of progression of stenosis [4]. An emergent endovascular recanalization may be required for a serious case with a concomitant intracranial large vessel occlusion if the patient meets the adequate indication at an early time window. If the patient is hemodynamically stable, a medical treatment can be the first option, but a close monitoring is required to ensure that there is a sufficient response to anticoagulation or antiplatelet therapy without recurrent ischemia. If the dissection is unstable, causing recurrent embolic or hemodynamic infarctions as in this case study, a carotid stenting is required to stabilize the lesion unless prompt and full responses to the medication is evidenced. Since only a few case reports for isolated spontaneous CCA dissection treated with a carotid stent can be found in the literature so far, further large-scale studies with long-term follow-up data are required to elucidate its feasibility and durability [4]. According to reports on CCA and ICA dissection caused by different etiologies, multiple stents were frequently required due to the long length of the dissection [4,7,8,12,13]. As in this case study, telescoping multiple stents can also be a feasible option to treat a long lesion of isolated spontaneous CCA dissection. Since tapered stents are most commonly used for the majority of carotid stenosis cases in recent years, large stents that fit into the usual size of the CCA may not be readily available commercially, and preprocedural arrangements, including an approximate measurement of the lesion size in advance and a preorder of non-tapered stents of various sizes, are important.

## Conclusions

An isolated spontaneous CCA dissection is an extremely rare disease, but it is an important cause of life-threatening strokes. High level of suspicion and detailed inspection of imaging are mandatory to prevent a misdiagnosis since a co-existing aortic dissection or a history of direct trauma is absent. Although there are no standardized guidelines for the treatment, carotid stent can be a feasible treatment option for unstable cases with recurrent ischemia. Telescoping multiple stents enables to stabilize a long-segment dissection.
